# High Homology-Directed Repair Using Mitosis Phase and Nucleus Localizing Signal

**DOI:** 10.3390/ijms21113747

**Published:** 2020-05-26

**Authors:** Jeong Pil Han, Yoo Jin Chang, Dong Woo Song, Beom Seok Choi, Ok Jae Koo, Seung Youn Yi, Tae Sub Park, Su Cheong Yeom

**Affiliations:** 1 Graduate School of International Agricultural Technology, Seoul National University, 1447 Pyeongchang-Ro, Daewha, Pyeongchang, Kangwon 25354, Korea; pil1426@snu.ac.kr (J.P.H.); jennayj917@gmail.com (Y.J.C.); taesubpark@snu.ac.kr (T.S.P.); 2 Department of Pharmacology, Yonsei University College of Medicine, 50 Yonsei-Ro, Seodaemun-gu, Seoul 03722, Korea; 3 Toolgen Inc., Gasan Digital-Ro, Geumcheon, Seoul 08594, Korea; dw.song@toolgen.com (D.W.S.); bs.choi@toolgen.com (B.S.C.); oj.koo@toolgen.com (O.J.K.); 4 NICEM PyeongChang branch, Greenbio Science and Technology, Seoul National University, 1447 Pyeongchang-Ro, Daewha, Pyeongchang, Gangwon 25354, Korea; yisy1@snu.ac.kr; 5 Designed Animal and Transplantation Research Institute, Greenbio Science and Technology, Seoul National University, 1447 Pyeongchang-Ro, Daewha, Pyeongchang, Gangwon 25354, Korea

**Keywords:** CRISPR, embryo, homology-directed repair, mitosis, NLS, ssODN

## Abstract

In homology-directed repair, mediated knock-in single-stranded oligodeoxynucleotides (ssODNs) can be used as a homologous template and present high efficiency, but there is still a need to improve efficiency. Previous studies have mainly focused on controlling double-stranded break size, ssODN stability, and the DNA repair cycle. Nevertheless, there is a lack of research on the correlation between the cell cycle and single-strand template repair (SSTR) efficiency. Here, we investigated the relationship between cell cycle and SSTR efficiency. We found higher SSTR efficiency during mitosis, especially in the metaphase and anaphase. A Cas9 protein with a nuclear localization signal (NLS) readily migrated to the nucleus; however, the nuclear envelope inhibited the nuclear import of many nucleotide templates. This seemed to result in non-homologous end joining (NHEJ) before the arrival of the homologous template. Thus, we assessed whether NLS-tagged ssODNs and free NLS peptides could circumvent problems posed by the nuclear envelope. NLS-tagging ssODNs enhanced SSTR and indel efficiency by 4-fold compared to the control. Our results suggest the following: (1) mitosis is the optimal phase for SSTR, (2) the donor template needs to be delivered to the nucleus before nuclease delivery, and (3) NLS-tagging ssODNs improve SSTR efficiency, especially high in mitosis.

## 1. Introduction

Eukaryotic cells have two DNA repair systems, namely error-prone non-homologous end joining (NHEJ) and error-free homologous recombination (HR). Traditionally, HR was used for gene targeting with embryonic stem cells, but random genetic damage caused by electroporation results in low specificity and efficiency. Recently developed nucleases can recognize specific sequences and create double-stranded breaks (DSBs). Zinc finger nucleases and transcriptional activator-like effector nucleases can bind to particular sequences but cannot create DSBs without conjugating to *Fok1*. Conversely, clustered regularly interspaced short palindromic repeat (CRISPR)/CRISPR-associated (Cas) nucleases can recognize complementary sequences and induce DSBs by themselves. These nucleases are widely utilized for gene manipulation using non-homologous end joining (NHEJ) or homology-directed repair (HDR) [1].

Single-stranded oligodeoxynucleotides (ssODNs) are used as a template for small sequence knock-in (KI) or nucleotide substitution. Traditional HR is a Rad51-dependent pathway that uses double-stranded DNA (dsDNA) as a template, while single-stranded template repair (SSTR) is Rad51-independent and uses the Fanconi anemia (FA) DNA repair pathway [2]. There have been many studies to improve SSTR efficiencies, such as 3′-end phosphorothioate or methyl-CpG modification of ssODNs [3,4], HR-promoting or NHEJ-blocking small molecules [5], and the use of overlapping single-guide RNAs (sgRNAs) [6]. Previous studies have mainly focused on the control of DSB size, ssODN stability, and the DNA repair cycle. Nevertheless, there is a lack of research on the correlation between cell cycle and SSTR efficiency. 

The cell cycle plays an essential role in repair pathway choice (NHEJ or HR) following DNA damage. For example, error-free HR mainly occurs during the S/G2 phases [7,8]. Even though SSTR and HR involve different mechanisms [2], increasing evidence suggests the cell cycle may be a potential regulator of SSTR efficiency. Recent studies have reported high SSTR efficiency during the G2/M phases [9,10]. However, this is different from our expectation that error-free repairs will take place during the S/G2 phases. Even though the cell cycle seems to be related to SSTR efficiency, cell experiments have limitations such as the difficulty of distinguishing the changes that occur in nuclear DNA. Mouse embryos have several advantages for cell cycle analysis to SSTR efficiency, including easy synchronization, scheduled superovulation, and a relatively large nucleus.

In this study, we investigated the correlation between the cell cycle and SSTR efficiency using mouse embryos. After defining the embryonic cell cycle, we electroporated Cas9 ribonucleoprotein (RNP) and ssODNs into mouse embryos. The mechanism behind cell-cycle-dependent changes in SSTR efficiency was identified and used to develop a method to improve SSTR efficiency.

## 2. Results 

### 2.1. Embryonic Cell Cycle Analysis by Nuclear Envelope Morphology

**Figure 1:**
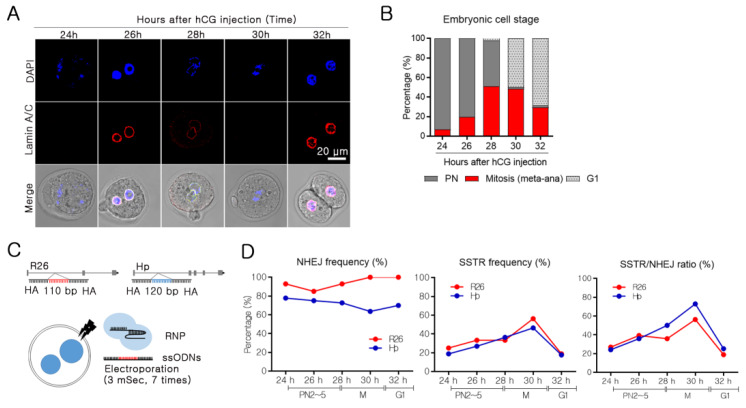
Identification of embryonic cell cycle, and cell cycle correlation with non-homologous end joining (NHEJ) and single-strand template repair (SSTR) in mouse embryos. (**A**) For whole-mount staining of the nuclei (DAPI) (blue) and lamin A/C (red). Randomly divided embryos were used for immune staining at 24 h, 26 h, 28 h, 30 h, and 32 h after human chorionic gonadotropin (hCG) injection. Fluorescence was detected using a confocal microscope. The embryonic cell stage was defined based on the presence of a nuclear envelope, nuclear condensation, and embryonic division. A representative image is shown for each time point. (**B**) The embryonic cell stage was defined, and the frequency was calculated. Embryos with two pronuclei were designated to PN, embryos with condensing nuclei and without a nuclear membrane were considered as mitosis, and two-cell embryos were considered to be in G1 (Number of analyzed embryos after 24 h: 45, 26 h: 41, 28 h: 45, 30 h: 58, and 32 h: 44). (**C**) Schematic representation of small sequence insertion using single-stranded oligodeoxynucleotides (ssODNs) (target loci: *Rosa26* and *Haptoglobin* (*Hp*)). For small sequence insertions of 110–120 bp into the target site, two binding site-overlapping sgRNAs and ssODNs with 40–45 nucleotides homology sequences were applied. (**D**) NHEJ frequency was calculated by PCR and T7E1 analysis after SpCas9 ribonucleoprotein (RNP) electroporation into embryos at each time point. The presence of SSTR was analyzed by single embryo PCR after SpCas9 RNP and ssODN electroporation, and the frequency was calculated as the % of KI embryos per total embryos.

Embryonic development has been extensively studied [11]; however, the cell cycle phases of early-stage embryos remain to be defined. Thus, we sought to identify the cell cycle of early-stage embryos at various times after human chorionic gonadotropin (hCG) injection. After estrus synchronization and superovulation, female mice were mated with sperm donor mice, and the embryos were collected 20 h later. As two-cell embryos develop at around 30 h after hCG injection, embryonic cell cycle analysis was performed between 24 and 32 h after hCG injection. The cell cycle of eukaryote consists of gap 1 (G1), synthesis (S), gap 2 (G2), and mitosis. However, early-stage embryos progress in the following order: pronuclear formation (PN 0~5), mitosis, and G1 [12]. The PN stage is further subdivided and is classified from PN1 to early PN3 as G1 phase, late PN3 to PN4 as S phase, and PN5 as G2 phase [13]. The embryonic cell cycle is defined by the presence of a nuclear envelope, chromosome condensation, and DNA synthesis [14]. Approximately 24 h after hCG injection, embryos were found to be in the PN2 stage. It was identified through the formation of a nuclear envelope with positive staining of lamin A/C and lamin B. Between 28 and 30 h after hCG injection, almost half of the embryos were found to be in the mitotic phase, characterized by chromatin condensation and loss of the nuclear envelope. Although the duration of mitosis was unclear, it appeared to last ~2 hours (Figure 1A and Figure S1, Supplementary Materials). The frequency of the mitotic embryos gradually increased until 28 h after hCG injection, and about half of the embryos were found to be in the mitotic phase between 28 and 30 h after hCG injection. Two-cell embryos (G1) were the dominant type at 32 h after hCG injection (Figure 1B). The embryonic cell cycle at each time point was similar to that reported by a previous study [12].

### 2.2. Mitosis-Specific High SSTR Efficiency 

After determining the embryonic cell cycle phase at each time point, two different target genes were used to investigate the correlation between the cell cycle and SSTR efficiency. One or two time points were chosen in PN, mitosis, and G1 phases. Next, 110 and 120 bp KI SSTR experiments were performed using the mouse embryos (Figure 1C). In electroporation with RNPs and ssODNs, T7E1 analysis indicated that the incidence of indel formation was approximately 80% in the early embryonic stage. Although the overall NHEJ frequency differed depending on the gene, there was a high frequency of DSB formation throughout the entire period (Figure 1D and Figure S2, Supplementary Materials). Interestingly, SSTR efficiency appeared to be correlated with the cell cycle, increasing to 40%~60% at mitosis, before decreasing again at G1 of the two-cell phase. It suggests a high SSTR efficiency in mitosis (Figure 1D and Figure S3, Supplementary Materials). High SSTR ratio to NHEJ in mitosis occurred, indicating that factors other than DSB formation were associated with high SSTR in mitosis.

### 2.3. The Nuclear Envelope as A Major Barrier to the Nuclear Import of ssODNs

**Figure 2:**
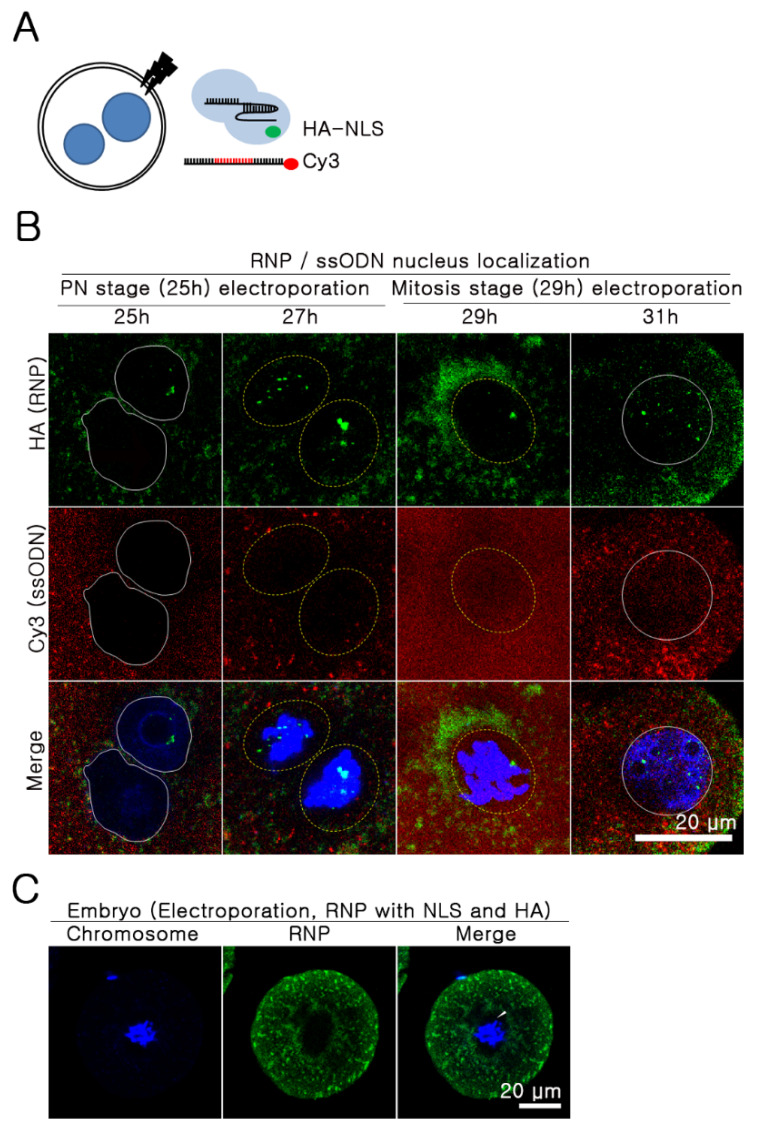
Nuclear localization of RNP and ssODN. (**A**) Experimental design. (**B**) RNP (HA-conjugated SpCas9 and sgRNAs targeting *Rosa26* locus) and Cy-3-conjugated 100 bp-sized ssODN were electroporated into embryos at 25 h and 29 h after hCG injection. Next, half were immunostained with HA-Alexa 488 (green) mAb, and the other half were stained after two hours with the same target. Representative images are shown. The related videos are presented in Videos 1 and 2, Supplementary Materials. White solid line: intact nuclear membrane; white dotted line: disappeared nuclear membrane. (**C**) Physical barriers outside the chromosome (white arrow); blue: chromosome; red: ssODN; green: RNP.

The most prominent features of mitosis are chromosome condensation and the loss of the nuclear envelope. Mitosis is subdivided into prophase, prometaphase, metaphase, anaphase, and telophase. Disappearance of the nuclear envelope was observed between metaphase and anaphase. Although chromosome condensation was expected to inhibit the access of RNPs and ssODNs to the target sequence, we focused on the correlation between the nuclear envelope and SSTR efficiency. All cell cycles were found to have a high indel frequency but varying SSTR efficiencies according to the phase of cells (Figure 1C,D), which suggests that there are different efficiencies of nuclear import of RNPs and ssODNs. To test this hypothesis, we prepared a 5′-Cy3-conjugated 100-nucleotide (nt) ssODN and a *Rosa26* locus-specific RNP, of which SpCas9 protein was conjugated to hemagglutinin and a nuclear localization signal (NLS). These allowed fluorescent detection of the localization of ssODN and RNP (Figure 2A). Electroporation was performed with embryos in PN and M phases, taking into account the presence of a nuclear envelope and the different efficiency of transportation. Half of the embryos were fixed immediately, and the remaining were fixed after a two-hour incubation to analyze changes over time. In the PN phase (25 h after hCG injection), RNPs crossed the nuclear envelope (green spots in the nucleus), but no ssODNs were translocated (red spots). Even at 2 h post-electroporation, more RNPs were found compared to ssODNs in the nucleus. On the other hand, both RNPs and ssODNs were diffusely distributed throughout the embryo, as there was no nuclear envelope at the mitotic phase (29 h after hCG injection). This result suggests that the lack of ssODNs as a homologous template during DNA cleavage by RNPs caused predominant NHEJ in the PN phase (Figure 2B, Videos 1 and 2, Supplementary Materials). The localization analysis of RNPs and ssODNs also exhibited several interesting findings. First, there appeared to be an immediate penetration of RNPs via an active nuclear envelope transport system using tagged NLS peptide (green spots in the nucleus, Figure 2B). Second, even though the structural nuclear envelope disappears during mitosis, there appear to be additional physical barriers outside the chromosome that restrict access of RNPs and ssODNs (Figure 2C and Figure S4, Supplementary Materials). 

### 2.4. High SSTR Efficiency in Mitotic Synchronous Embryos and Cells

**Figure 3:**
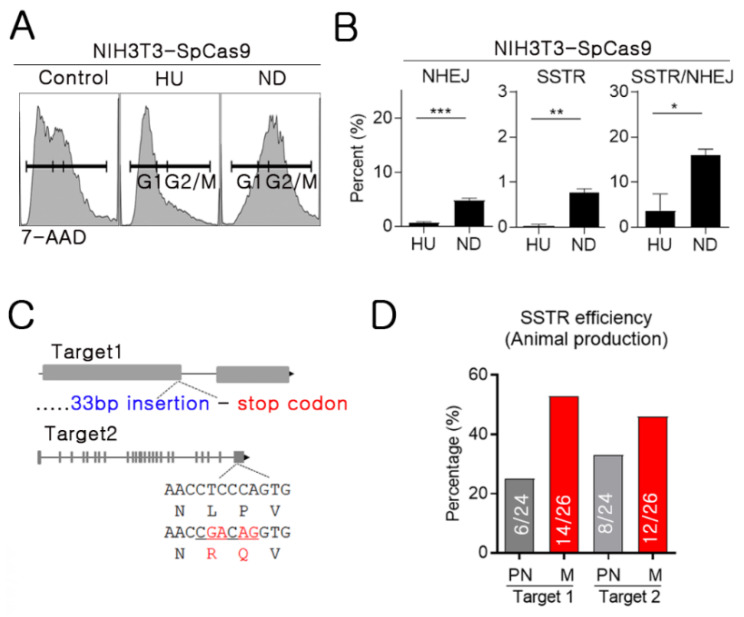
NHEJ and SSTR analysis throughout the cell cycle. (**A**) Confirming cell cycle synchronization using flow cytometry of hydroxyurea- and nocodazole-treated cells. Anti-7-AAD was used for detection. HU: hydroxyurea, ND: nocodazole. (**B**) NHEJ and SSTR efficiency were calculated using data from deep sequencing-based analysis (% of NHEJ or KI reads / total reads). (**C**) Mouse generation with SSTR strategy; 33 bp insertion (Target 1) and nucleotide alteration (Target 2) (**D**) SSTR efficiency was calculated (% of KI pups per total produced pups). Grey bar: electroporation at PN (3 p.m., 25 h after hCG injection). Red bar: electroporation at mitosis (7 p.m., 29 h after hCG injection).

Next, we used the NIH3T3 with SpCas9 overexpression cell line (NIH3T3-SpCas9) to confirm the high SSTR efficiency in mitosis. We selected nocodazole to block in the G2/M phase and hydroxyurea to block in G1/S based on established protocols for cell cycle synchronization [9] (Figure 3A). In the deep sequencing-based analysis, the nocodazole-treated group showed significantly higher NHEJ and KI rates than the hydroxyurea-treated group (Figure 3B). The SSTR ratio to NHEJ was higher in the nocodazole-treated group (G2/M phase), suggesting that the G2/M phase provides better conditions than G1 for nuclear import of the RNPs and ssODN templates. We utilized NIH3T3-SpCas9 on every experimental group of cell experiments. Since there is no direct interaction between Cas9 protein and ssODN, NIH3T3-SpCas9 would not influence SSTR efficiency. To confirm the reproducibility of high SSTR efficiency in the mitotic phase, we compared KI efficiency on animal production. Target mutations were a 33 bp insertion before the stop codon for in vivo gene expression analysis [15] and with nucleotide alteration, which was found in a human patient (four-base alteration) (Figure 3C). A simple comparison of KI animal production efficiency in PN and mitosis was conducted using electroporation at two different time points: 25 h (PN) and 29 h after hCG injection (mitosis). The result of the PN phase electroporation showed a high SSTR efficiency of 25% and 33%. However, mitotic electroporation showed a 10%–20% higher SSTR rate of 54% and 46% (Figure 3D and Figure S5, Supplementary Materials). Overall, the mitosis phase appears to be the appropriate time to increase SSTR in cells and embryos.

### 2.5. Minimal Impact of ssODN Size and Cdk1 on SSTR Efficiency

**Figure 4:**
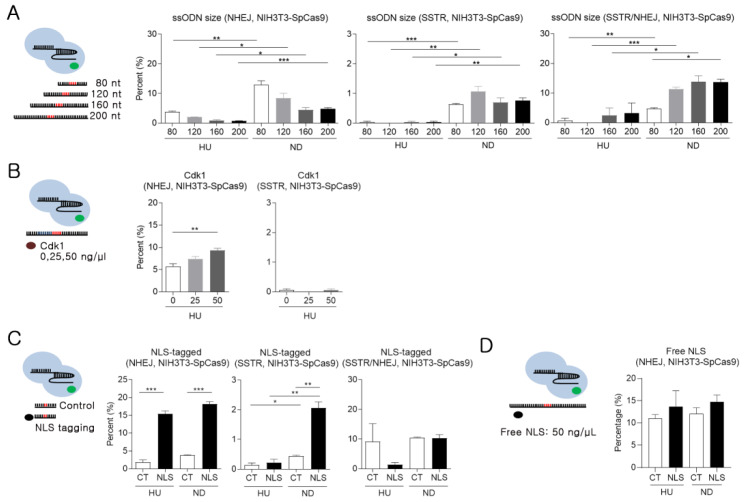
NHEJ and SSTR analysis of modified ssODNs and protein addition. (**A**,**C**) The efficiency of NHEJ, SSTR, and SSTR/NHEJ ratio was analyzed using the different-sized ssODNs or NLS-tagged ssODNs. (**B**,**D**) NHEJ frequency calculated on Cdk1 or free NLS peptide treatment. Red region: 20 bp KI sequence. Black region: homologous sequence. HU: hydroxyurea, ND: nocodazole. Statistical analysis was performed using Student’s *t*-test. *: *p* < 0.05, **: *p* < 0.01, and ***: *p* < 0.001.

Cell cycle synchronization and cell cycle-specific gene editing seem to be the most straightforward approaches for highly efficient HDR. However, nocodazole could raise potential limitations, such as erythrocyte toxicity [16]. Thus, further experiments were conducted to develop efficient transport methods that circumvented the nuclear envelope. As such, we first assessed the efficiency of transport mediated by passive uptake using ssODNs of varying lengths (80 and 200 bp). Nocodazole-treated cells (G2/M) still displayed significantly higher NHEJ frequency than hydroxyurea-treated cells, and smaller ssODNs exhibited higher NHEJ frequencies than longer ssODNs in both the nocodazole- and hydroxyurea-treated cells. However, the overall SSTR efficiency was similar between all the cells treated with ssODNs of different sizes (Figure 4A). Despite the low NHEJ rate with longer ssODNs, the longer length of the ssODN homology arm was allocated, which resulted in a higher SSTR efficiency compared to that of other groups. Since the SSTR/NHEJ ratio was higher when using long ssODNs, their use seems to be a more effective strategy in SSTR experiments. After confirming that ssODN size does not enhance SSTR efficiency, we examined the SSTR frequency on physical changes of the nuclear envelope. As Cdk1 and cyclin B control the disassembly of the nuclear envelope in mitosis [17], we compared the NHEJ and SSTR efficiency of G1-synchronized NIH3T3-SpCas9 cells after Cdk1 protein treatment. The results of the Cdk1 protein treatment (0, 25, and 50 ng/µL) confirmed a dose-dependent increase in NHEJ frequency, but SSTR efficiencies were similar between other groups (Figure 4B). 

### 2.6. Improving SSTR Efficiency Using NLS-tagged ssODNs

Next, we used NLS-tagged ssODNs to study active transportation via the nuclear pore complex (NPC). As RNPs were already NLS-conjugated, the NHEJ rates were predicted to occur at a similar rate in all the groups. However, NLS-tagged ssODNs increased the NHEJ rates in both the hydroxyurea and nocodazole groups. Furthermore, NLS-tagged ssODNs increased the SSTR efficiency by over 4-fold compared to the control in the nocodazole-treated cells. In particular, the SSTR/NHEJ ratio was different according to the cell cycle phase. NLS-tagged ssODNs showed relatively low SSTR/NHEJ values compared to those of the control in hydroxyurea treatment conditions (G1 phase). However, the SSTR/NHEJ ratio was similar between groups under nocodazole treatment conditions (G2/M phase) (Figure 4C). These results indicate that NLS-tagging ssODNs do not improve the nuclear import of template ssODNs, but rather increase NHEJ efficiency by increasing RNP access to the target site. In other words, the DSB frequency increased, but the nuclear access of ssODNs was still limited in the G1 phase; therefore, the SSTR/NHEJ ratio decreased. On the other hand, higher DSB rates of NLS-tagged ssODNs had a role in high SSTR efficiency using sufficient ssODNs around the DSB site in the G2/M phase. Similarly, free NLS peptides enhanced NHEJ efficiency in the SSTR experiment with NIH3T3-SpCas9 cell (Figure 4D). The mechanism(s) of NLS peptide-induced increases in NHEJ are unclear. However, NLS-tagged ssODNs seem to enhance the rate of RNP delivery throughout the cell cycle and increase SSTR efficiency, especially during mitosis.

## 3. Discussion

CRISPR/Cas9 has been used as a tool for genetic modification of the loss of function or gain of function in a variety of fields [18]. Gene editing efficiency is particularly important for cell therapy and in vivo gene correction, and various methods for enhancing HDR efficiency have been studied. Previous studies have reported on the association of the cell cycle with HDR efficiency; they suggested that high HDRs could be induced through cell cycle synchronization using nocodazole or cell division cycle-related protein kinase [9,10,19]. However, there were no reports on the interaction or mechanism between HDR efficiency and intracellular structures across the cell cycle. Our study demonstrates that the cell cycle plays an essential role in HDR efficiency, especially in the restriction of the transport of DNA templates by the NPC. Moreover, NLS-tagging is proven to be a viable method for enhancing NHEJ and SSTR efficiency.

Mouse embryos have been used to study the cell cycle [12,14]. Here, to analyze gene-editing efficiency across the cell cycle, we defined the embryonic cell cycle at several time points of fertilized mouse embryos using a morphological analysis of chromosomes and the nuclear membrane. Because we used in vivo fertilized embryos, the cell cycle of each time point was not homogenous but heterogeneous. However, during mitosis (especially metaphase or anaphase), a higher SSTR efficiency was found, which was caused by the absence of a nuclear envelope. The FA DNA repair pathway is the DNA repair mechanism underlying SSTR [2]. It is activated by inter-strand crosslinks that occur at S phase [20], and it maintains chromosomal stability during DNA repair from G2 to M [21]. Even Fanconi anemia, complementation group M, as one of the main components of the FA pathway and an essential player in SSTR, is hyper-phosphorylated in mitosis [2,22]. The previous studies suggested that SSTR would actively occur in the G2/M phases. Therefore, high SSTR efficiency during mitosis is a consistent result. However, the effect of the FA pathway on high SSTR efficiency in mitosis has not been identified in this study.

To increase the SSTR efficiency, it was necessary to simultaneously deliver the RNPs and DNA templates to the target locus. In general, NLSs are fused to SpCas9 to facilitate their transfer into the nucleus; thus, it would deserve consideration to NLS-tag a DNA template for using active transportation [23]. Although the NLS-tagging of dsDNA did not increase nuclear localization [24], we analyzed it because there were no previous reports on the efficiency of SSTR using NLS-tagged ssDNA. Even though NLS-tagged ssODNs did not improve nuclear localization, we found that using NLS-tagged ssODNs improved the efficiency of NHEJ and SSTR, especially during mitosis. Furthermore, the simultaneous application of NLS-tagged ssODNs and nocodazole treatment could be a better method for enhancing HDR efficiency. Although the reason for the increase in NHEJ frequency with NLS-tagged ssODNs is unclear, NLS peptides may influence NPC activity, resulting in increased transport of RNPs to the target. Recently, several methods to improve SSTR efficiency, including peptide and oligo conjugation, have been proposed [25].

When it is not possible to utilize mitosis phase synchronization, DNA template delivery via NPC is necessary. For example, HR is predominant in the G2/S phase. The cells in this phase have an intact nuclear envelope, and high HR efficiency can be achieved when energy-dependent NPC transportation is carried out via linking RNPs with DNA templates [26]. Similarly, high SSTR efficiency has been induced by covalently binding RNPs and ssODNs [27,28]. One of the current applications of CRISPR/Cas9 is in vivo gene editing. In vivo HDR gene editing with adeno-associated virus (AAV) is not a problem as AAV transduces into the nucleus and synthesizes Cas9 protein after dsDNA formation [29]. However, when using a non-viral vector, such as lipid nanoparticles, the nuclear membrane should be efficiently controlled. Recently, several studies have improved nuclear DNA transport using supramolecules with acidity-accelerative decomposition or the NLS-tagging strategy [23,30].

In this study, we analyzed the relationship between the nuclear envelope and SSTR efficiency using mouse embryos and cells. Their applicability is expected to increase, including their use for in vivo gene editing. In summary, HDR efficiency is determined by the DSB potential of Cas9 and the simultaneous access of the donor template. For this, it is necessary to consider a strategy based on the phases of the cell cycle to deliver the donor template efficiently into the nucleus. 

## 4. Materials and Methods

### 4.1. Animal and Embryo Preparation

C57BL/6 mice were obtained from Koatech (PyeongTaek, Korea) and maintained under specific pathogen-free conditions. Female mice were injected with 5 IU of serum gonadotropin (Prospec Bio, East Brunswick, NJ, USA) and 5 IU of hCG (Prospec) at 48 h intervals for estrus synchronization and superovulation. The female mice were then mated with the sperm donor mice at 2 p.m. Embryos were collected from the oviducts 20 h later at 10 a.m. the next day, and were cultured in KSOM medium (Merck Millipore, Billerica, MA, USA). This study was approved by the Institutional Animal Care and Use Committees of Seoul National University (SNU-180315-4, SNU-1708164, and SNU-180827-4) and was conducted in accordance with recommended guidelines. 

### 4.2. Embryonic Whole-Mount Immunostaining for Cell Cycle Determination

The embryos were randomly divided into five groups. The whole-mount immunofluorescence of lamin A/C and lamin B was performed 24, 26, 28, 30, and 32 h after hCG injection. Briefly, the embryos were fixed in 4% paraformaldehyde in PBS for 30 min. Next, this was followed by washing four times in 0.01% bovine serum albumin in PBS (BSA-PBS), permeabilization with 0.5% Triton X in PBS for 30 min, and blocking with 3% BSA-PBS. Then, the embryos were incubated overnight at 4 °C with the primary antibodies lamin A/C Alexa 594 (red fluorescence and clone: E-1) (Santa Cruz Biotechnology, Dallas, TX, USA) and lamin B Alexa 488 (green fluorescence and clone: B-10) (Santa Cruz). The following day, the embryos were mounted on slides using DAPI-containing mounting gel (ProLong Gold antifade reagent; Invitrogen, Carlsbad, CA, USA). Fluorescence was detected using a confocal microscope (Leica TCS SP8; Leica, Wetzlar, Germany) the next day.

### 4.3. In silico sgRNA Design and Homologous Template Preparation

For this study, sgRNAs were designed with a 20 bp binding sequence to target the *Rosa26* locus and *Haptoglobin* (*Hp*), namely Targets 1 and 2. They were synthesized using an in vitro RNA synthesis kit (Thermo Fisher Scientific, Waltham, MA, USA) after PCR amplification. The sgRNA of each target region was designed with overlapping binding sites [6]. Moreover, 200 bp ssODNs were also prepared using commercial service (Integrated DNA Technologies, Skokie, IL, USA). The sgRNA and ssODN sequences are provided in Tables S1 and S2, Supplementary Materials. 

### 4.4. Electroporation-Mediated Ribonucleoprotein Transfection into Embryos and Genotyping

Electroporation was performed to transfer the Cas9 RNPs and ssODNs into the embryos at the five time points according to previously established conditions [31]. Briefly, the embryos were washed three times with Opti MEM I medium (Invitrogen, Carlsbad, CA, USA). Then, embryos per group were transferred into the electroporation buffer on the electrode. The final concentration of the electroporation buffer consisted of 200 ng/μL of *Streptococcus pyogenes* Cas9 protein (SpCas9) (Toolgen Inc, Seoul, Korea), 50 ng/μL of each sgRNA, and 100 μ mole of ssODN. For analyzing NHEJ efficiency, the RNP for each target was transfected into the embryo, while the RNP and ssODN mixture was applied in the embryo experiment for SSTT efficiency analysis. The electroporation pulse conditions were as follows: 7 cycles of 30 V with 3 ms ON and 97 ms OFF. After washing with M2 medium (MTI-GlobalStem, Rockville, MD, USA), the embryos were cultured under the KSOM medium (Merck Millipore, St. Louis, MO, USA) until performing embryo transfer or genotyping. For embryo transfer of Targets 1 and 2, about 20 embryos were transferred to the oviduct of recipient mice. For blastocyst genotyping of *Rosa26* and *Hp* targets, single embryos were transferred to 10 µL of distilled water and used as the PCR template after performing three freeze–thaw cycles and incubating at 95 °C for 15 min. Next, the PCR amplicons were subjected to an additional T7E1 assay (NEB, Ipswich, MA, USA) or gel running for detecting SSTR. For genotyping of the fetus (Targets 1 and 2), primers were designed, and SSTR of all targets was defined by the existence of target-sized amplicons. All primers used for genotyping are listed in Table S3, Supplementary Materials. 

### 4.5. Nuclear Localization Analysis for Cas9 Protein and ssODNs

A 100 bp ssODN was synthesized with a Cy-3 peptide conjugation form at the 5′ end (Bioneer, Daejeon, Korea). The embryos were randomly divided into two groups. Next, this was followed by electroporation with hemagglutinin (HA)-conjugated SpCas9 RNP with *Rosa26* targeting sgRNAs and Cy3-conjugated ssODN at 25 h (3 p.m.) and 29 h (7 p.m.) after hCG injection. Half of the embryos were immediately fixed with 4% paraformaldehyde in PBS, and the remaining embryos were fixed after two hours. Embryo whole-mount immunostaining was performed using anti-HA Alexa 488, to detect RNPs (green fluorescence), and DAPI. Fluorescence was observed using a confocal microscope (Leica), and the localization of the green and red signals was analyzed (Figure 3A). 

### 4.6. Establishing SpCas9 Overexpression NIH3T3 Cells

To develop SpCas9 overexpression (NIH3T3-SpCas9) cells, three µg of pITR-CAG-SpCas9/RF)-ITR and pPiggyBac transposase were co-transfected into 6 × 10^5^ NIH3T3 cells (ATCC CRL-1658; American Type Culture Collection, Manassas, VA, USA). Three days after transfection, high RFP-expressing cells were sorted by flow cytometry. 

### 4.7. Preparation of Modified ssODNs and Cdk1

For the preparation of NLS-tagged ssODNs, 60-nucleotide-sized ssODNs with 25 bp homology and 10 bp KI sequence were synthesized. Half of these were then tagged with an SV40-derived nuclear localization signal peptide (Pro-Lys-Lys-Lys-Arg-Lys-Val-Cys)(Genscript, Piscataway, NJ, USA). Additionally, ssODNs of different sizes were designed and synthesized as 20 bp of a KI sequence with 30, 50, 70, and 90 bp homologous sequences (Integrated DNA Technologies, Coralville, IA, USA). The sequences of each ssODN are provided in Table S2, Supplementary Materials. Cdk1 (Sigma, St. Louis, MO, USA) and NLS peptide (Genscript) were purchased by commercial service. 

### 4.8. Cell Cycle Synchronization and Transfection of Cells by Electroporation

The NIH3T3-SpCas9 cell line was cultured in DMEM and 10% FBS. To synchronize the cells in phases G1 and G2/M, 2 mM of hydroxyurea (Sigma) and 200 ng/mL nocodazole (Sigma) were added to 1 × 10^6^ of cells [9]. The cell cycle was then analyzed using 7-aminoactinomycin D (7-AAD) stain and flow cytometry analysis (BD Bioscience, San Jose, CA, USA). Next, transfection into 2 × 10^5^ NIH3T3-SpCas9 with a mixture containing 1 μg of *Rosa26* locus-targeting sgRNA and 100 μ mole ssODN was performed using a Neon electroporator (Thermo Fischer Scientific). Cell harvesting and gDNA extraction were performed 72 h after transfection, followed by deep sequencing.

### 4.9. Targeted Deep Sequencing 

After performing PCR using Phusion Taq polymerase (New England Biolabs, Ipswich, MA, USA), the PCR amplicons were subjected to paired-end deep sequencing using Mi-seq (Illumina, San Diego, CA, USA). The deep sequencing data were analyzed using Cas-Analyzer (www.rgenome.net) [32]. An indel appearing 3 bp upstream of the ‘5-NGG-3’ PAM was considered as a mutation caused by RNP, and KI efficiency was calculated with matching between read sequence and the reference sequence. 

### 4.10. Statistical Analysis

Statistical analysis was performed using unpaired Student’s *t*-test with GraphPad Prism (version 5.02, GraphPad, San Diego, CA, USA). Significance was defined as *p* < 0.05.

## Supplementary Materials

The following are available online at https://www.mdpi.com/1422-0067/21/11/3747/s1.
