# Supplementary material for: High Homology-Directed Repair Using Mitosis Phase and Nucleus Localizing Signal
Source: Int J Mol Sci. 2020 May 26;21(11):3747. doi: 10.3390/ijms21113747 (PMC7312558; doi:10.3390/ijms21113747)
Supplement: Supplementary file 1 [file ijms-21-03747-s001.zip › ijms-664609-supplementary.docx]

Supplementary information for

**High homology directed repair using mitosis phase and nucleus localizing signal**

**Running title:** High HDR with mitosis and NLS

**Authors**

Jeong Pil Han^1^, Yoo Jin Chang^1,2^, Dong Woo Song^3^, Beom Seok Choi^3^, Ok Jae Koo^3^, Seung Youn Yi^4^, Tae Sub Park^1^, Su Cheong Yeom ^1,5*^

**Corresponding authors**

**Su Cheong Yeom**, DVM, Ph.D., Associate professor, Graduate School of International Agricultural Technology, Seoul National University, 1447 Pyeongchang-Ro, Daewha, Pyeongchang, Gangwon 25354, Korea

Tel: 82-33-339-5750, Fax: 82-33-339-5762, E-mail: scyeom@snu.ac.kr

This file includes

1. Whole-mount stained embryo with Lamin A/C, Lamin B and RNP
2. Electrophoresis images of PCR and T7E1 for Rosa26, HP, Target 1, and 2.
3. Sequences information of sgRNAs, ssODNs and primers


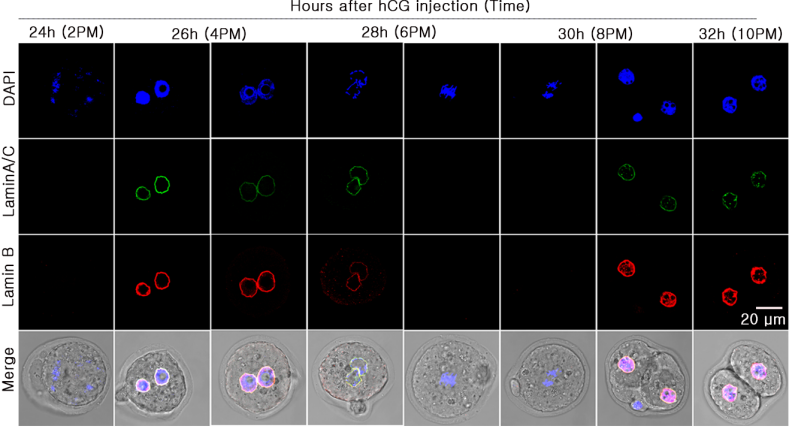


**Supplementary figure 1. Identification of embryonic cell cycle.** For whole-mount staining of the nuclei (DAPI) (blue), Lamin B (green), and Lamin A/C (red), randomly divided embryos were used for immune staining at 2, 4, 6, 8, and 10 pm. Fluorescence was detected using a confocal microscope. A representative image is shown for each time.

**Supplementary figure 2.** Electrophoresis image of T7E1 for Rosa26 and Hp targets. Yellow symbols indicate NHEJ (T7E1 positive). PCR for Rosa26 target was conducted using Rosa26-1 primer set (Detail primer sequences were in the supplementary table 3)

**Supplementary figure 3.** Electrophoresis image of SSTR for Rosa26 and Hp targets, Yellow symbols indicate KI. PCR for Rosa26 target was conducted using Rosa26-2 primer set (Detail primer sequences were in the supplementary table 3)


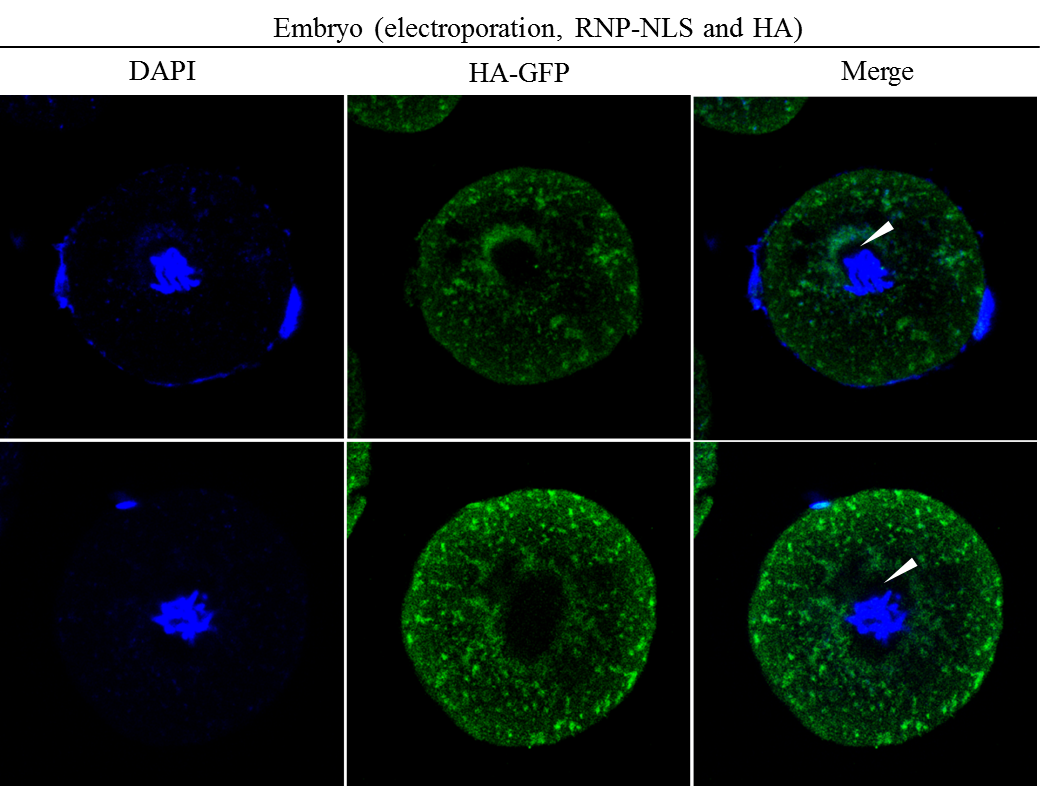


**Supplementary figure 4.** The physical barrier around the condensed chromosome in mitosis. Blue: Nuclei (DAPI, Green spot: HA conjugated RNP


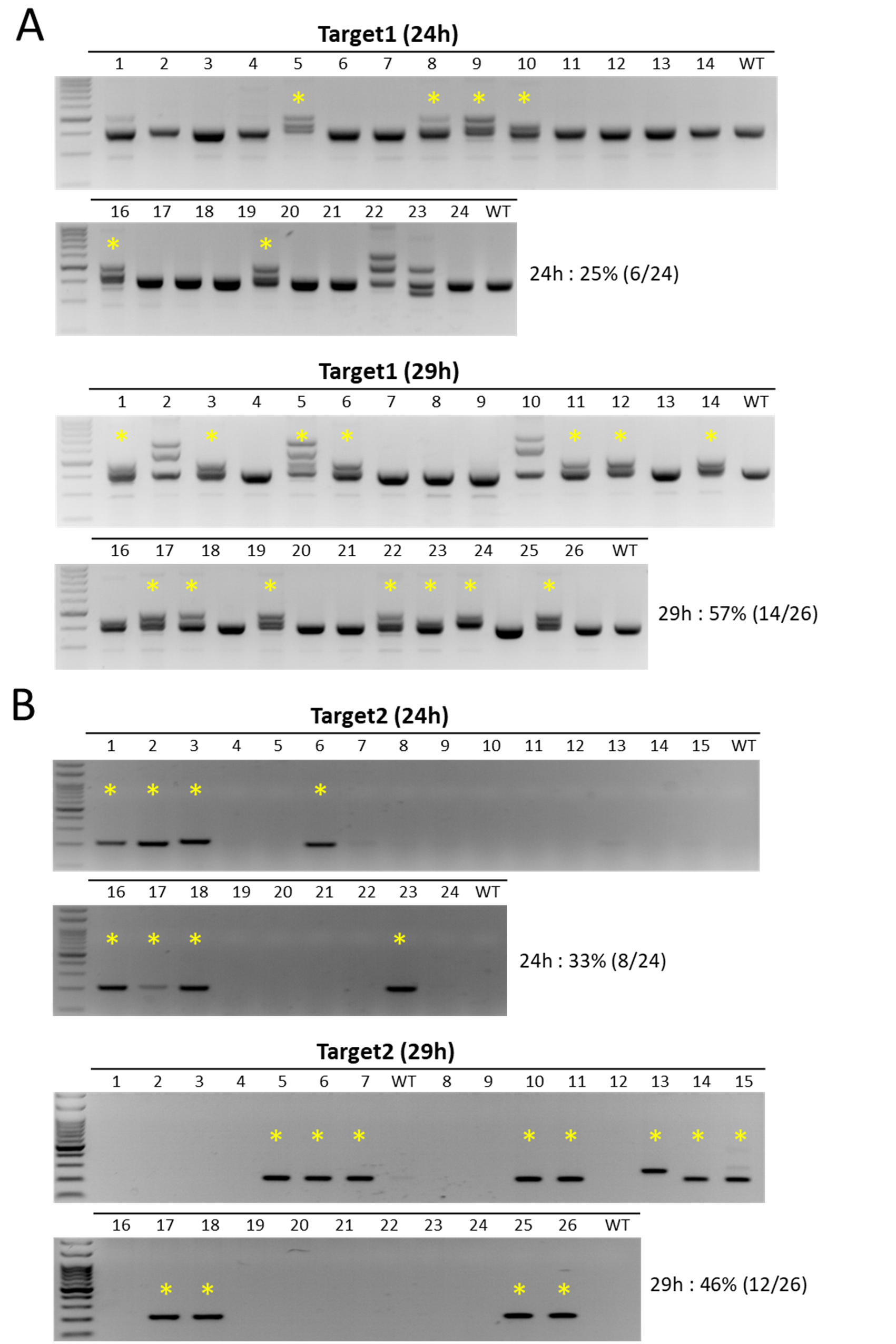


Figure 5. Electrophoresis image of SSTR for target 1 and 2, Yellow symbols indicate KI.

Supplementary Table 1. sgRNA sequences used in this study

| Target gene |  | sgRNA binding sequence | PAM |
| --- | --- | --- | --- |
| *Rosa26* | sgRNA 1 | GACTGGAGTTGCAGATCACG | AGG |
|  | sgRNA 2 | gcagatcacgagggaagagg | GGG |
| *Hp* | sgRNA 1 | ccaatgatggccacagtcat | AGG |
|  | sgRNA 2 | gatggccacagtcataggtt | Agg |
| *Target1* | sgRNA1 | cagggttttcagctcctaac | tgg |
|  | sgRNA2 | tggggcgggaggatccagtt | agg |
| *Target2* | sgRNA1  sgRNA2 | cactgggaggttatcactgg  agtccgacgcacgcactggg | tgg  agg |

Supplementary Table 2. ssODN sequences for genotyping used in this study

| Target gene | sgRNA binding sequence |
| --- | --- |
| *Rosa26* | GGACCGCCCTGGGCCTGGGAGAATCCCTTCCCCCTCTTCCCTCGTATAACTTCGTATAATGTATGCTATACGAAGTTATTTCGAATTCTGCAGTCGACGGTACCGCGGGCCCGGGATCCATATAACTTCGTATAGGATACTTTATACGAAGTTATGATCTGCAACTCCAGTCTTTCTAGAAGATGGGCGGGAGTCTTCTG |
|  |  |
| *Hp* | AGCATTTAAGTGTGGTCTGTGTACCAATGATGGCCACAGTAGTTATCTACATTTTATTACCACTATCTTTGCGGTGTCTGAGGGAGGTTTCTCTTTCCTGGAGGGCTCCTGTATTATTGCCAATGTACTTTCCTGAATGCAGCCAGAAACTGAGCCCACCCATAGGTTAGGAAGACAGACAGTTTTCTGGCATTCTTGGG |
|  |  |
| *Rosa26-NLS* | GAATCCCTTCCCCCTCTTCCCTCGTAATGCGTACCGATCTGCAACTCCAGTCTTTCTAGA |
| *Rosa26-60nt* | TGGGAGAATCCCTTCCCCCTCTTCCCTCGTGGATTATTCATACCGTCCCAGATCTGCAACTCCAGTCTTTCTAGAAGATG |
| *Rosa26-120nt* | TCTGAGGACCGCCCTGGGCCTGGGAGAATCCCTTCCCCCTCTTCCCTCGTGGATTATTCATACCGTCCCAGATCTGCAACTCCAGTCTTTCTAGAAGATGGGCGGGAGTCTTCTGGGCAG |
| *Rosa26-160nt* | TTCTCTGCTGCCTCCTGGCTTCTGAGGACCGCCCTGGGCCTGGGAGAATCCCTTCCCCCTCTTCCCTCGTGGATTATTCATACCGTCCCAGATCTGCAACTCCAGTCTTTCTAGAAGATGGGCGGGAGTCTTCTGGGCAGGCTTAAAGGCTAACCTGGTG |
| *Rosa26-200nt* | TTGCAATACCTTTCTGGGAGTTCTCTGCTGCCTCCTGGCTTCTGAGGACCGCCCTGGGCCTGGGAGAATCCCTTCCCCCTCTTCCCTCGTGGATTATTCATACCGTCCCAGATCTGCAACTCCAGTCTTTCTAGAAGATGGGCGGGAGTCTTCTGGGCAGGCTTAAAGGCTAACCTGGTGTGTGGGCGTTGTCCTGCAGG |
| *Target1* | CCCTGACCCCCGAAGAAAGCTAAGGAACCTGGTTTCCACGCCACTAACACCTTCTCTTGTCCAGCAGGGTTTTCAGCTCCGTGAGCGGCTGGCGGCTGTTCAAGAAGATTAGCTAACTGGATCCTCCCGCCCCATGGGATATGGGGTGGCTGCCCTGAGTGCCCACGCGAGGGCGGCCGCAAGTTGACTAAAC |
|  |  |
| *Target2* | CCCTCTCCAGCTCTTCCATCTCCTCCACGTCCTTCCCACCGTCCTATGACAGCGTCACGAGGGCCACCAGTGATAACCTCCCAGTGCGTGCGTCGGACTACAGCCGCAGCGAAGATCTTGCAGACTTCCCTCCATCTCCAGATAGGGACCGAGAGTCTAT |

Red alphabets: KI sequences, Black alphabets: homology arm sequences

Supplementary Table 3. Primer sequences for genotyping used in this study

| Target gene | Primer | Product size (bp) | Annealing Tm (ºC) |
| --- | --- | --- | --- |
| *Rosa26-1* | F: 5’-GTTGGGCTGTTTTGGAGG-3’ | WT: 641, KI: 751 | 60 |
|  | R: 5’-GGAGCGGGAGAAATGGAT-3’ |  |  |
| *Rosa26-2* | f: 5’-ggaggggagtgttgcaat-3’ | wt: 146, ki: 256 | 60 |
|  | r: 5’-cagaagactcccgcccatct-3’ |  |  |
| *Hp* | F: 5’-TACGGGGAGAGGTGAGAAG-3’ | WT: 787, KI: 907 | 60 |
|  | R: 5’-GTCATAGTCGCTGTTGTTGT-3’ |  |  |
| *Target1* | F: 5’-TGGAGAGTTAGGGGCAGT-3 | WT: 423, KI: 457 | 61 |
|  | R: 5’-GGGTTGGGGTTGAGAGAAG-3 |  |  |
| *Target2* | F: 5’-GCATGCTTCCTTCCTCTT-3 | WT: no band, KI: 212 | 60 |
|  | R: 5’-CACGCACCTGTCGGTTAT-3 |  |  |
